# A Tissue Section-Based Near-Infrared Spectroscopical Analysis of Salivary Gland Tumors

**DOI:** 10.3390/cancers13215356

**Published:** 2021-10-26

**Authors:** Renaat Coopman, Sander De Bruyne, Marijn Speeckaert, Tijl Vermassen, Hubert Vermeersch, David Creytens, Joris Delanghe

**Affiliations:** 1 Department of Oro-Maxillofacial, Plastic, Reconstructive and Aesthetic Surgery, Ghent University Hospital, 9000 Ghent, Belgium; Renaat.Coopman@UGent.be (R.C.); Hubert.Vermeersch@UGent.be (H.V.); 2 Department of Diagnostic Sciences, Ghent University Hospital, 9000 Ghent, Belgium; SanderR.DeBruyne@UGent.be; 3 Department of Clinical Chemistry, Microbiology and Immunology, Ghent University, 9000 Ghent, Belgium; 4 Department of Nephrology, Ghent University Hospital, 9000 Ghent, Belgium; Marijn.Speeckaert@ugent.be; 5 Research Foundation-Flanders (FWO), 1000 Brussels, Belgium; 6 Department of Medical Oncology, Ghent University Hospital, 9000 Ghent, Belgium; tijl.vermassen@uzgent.be; 7 Department of Diagnostic Sciences and Cancer Research Institute Gent (CRIG), Ghent University, 9000 Ghent, Belgium; David.Creytens@UGent.be; 8 Department of Pathology, Ghent University Hospital, 9000 Ghent, Belgium

**Keywords:** infrared, spectroscopy, NIR, salivary gland tumors, tissue section

## Abstract

**Simple Summary:**

Salivary gland tumors (SGTs) are a group of rare tumors that vary in clinical and histological behavior. Histological classification is difficult and requires an experienced pathologist. Based on recent research in other medical fields, this study investigated the value of near-infrared (NIR) spectroscopy in the diagnosis of SGTs supplementary to the histological investigation. The acquired spectra were analyzed with chemometric techniques. Enzymatic treatment (neuraminidase) resulted in spectral peak differences between healthy controls and different SGT types. Some malignant SGTs had higher spectral changes, suggesting bigger alterations in glycosylation of salivary mucins. Future biochemical research based on the further enzymatic dissection of SGTs and infrared spectroscopy could help pathologists to better understand the nature of these types of tumors.

**Abstract:**

SGTs vary in histological behavior. Mucins, a major component in salivary glands, consist of a glycosylated and sialylated protein core. Rapid evolutions in glycobiology have demonstrated the important role of glycoproteins in cancer development. NIR spectroscopy is a method for the biochemical analysis of substrates. NIR spectra can be analyzed using specific chemometrics. Our aim was to explore the diagnostic possibilities of NIR spectroscopy in SGTs. 238 Hematoxylin and Eosine stained (H&E) SGT tissue sections were examined using NIR spectroscopy. 45 deparaffinized tissue sections were treated with neuraminidase to identify wavelengths in the NIR spectrum related to sialylation. NIR spectra were analyzed with chemometrics. NIR spectra could distinguish malignant SGTs from controls and benign SGTs. Prediction models based on the entire spectral range resulted in a 73.1% accurate classification of malignant SGTs and controls, while, based on neuraminidase experimental spectral peak differences (1436 nm; 1713 nm; 1783 nm; 1924 nm; 2032 nm; 2064 nm; 2178 nm; 2216 nm), an improved overall correct classification rate of 91.9% was obtained between healthy subjects and malignant tumors. H&E tissue section-based NIR spectroscopy can identify malignant SGTs from controls, promising an alternative method in the diagnosis of SGTs.

## 1. Introduction

SGTs are an uncommon, heterogeneous group of neoplasms that vary considerably in their anatomic site of origin, histology, and biologic behavior. Both benign and malignant SGTs are classified according to the World Health Organization (WHO) system of 2017 [1]. Anatomically, the parotid gland is the most frequent site of SGTs, accounting for approximately 80 to 85 percent of these tumors [2,3]. Histologically, the most common types of SGTs are pleomorphic adenoma (benign), Whartin tumor (benign), mucoepidermoid carcinoma (malignant), and adenoid cystic carcinoma (malignant). Around 80% of SGTs are benign, and 65% of these are pleomorphic adenoma, which are by far the most common of all SGTs. SGTs are very rare with reported incidences of only 1.2–1.3 cases per 100,000, representing only around 3% of all cancers of the head and neck [3].

Diagnosis of SGTs is based on histological investigation and starts with a microscopic analysis of the layered arrangement of three types of salivary cells: epithelial, myo-epithelial, or its stroma on H&E tissue sections. The proportion of each tissue layer varies within each SGT. Multiple immunostains (CK7, p63, S100, GFAP, HER2) were developed to ease the histological differentiation process [3,4]. However, pathologists are still often faced with difficulties in SGT diagnosis due to their rarity, resulting in a long learning curve [3].

In recent years, glycobiology has gained increased importance in cancer research because of the shift from the normal glycosylation pathway that occurs in cancer cells, resulting in altered glycan expression. One of the most widely occurring cancer-associated changes in glycosylation is sialylation. Through the process of sialylation, sialic acid is bound to oligosaccharides, which have an important role in cellular recognition, cell adhesion, and cell signaling. An increase in global sialylation has been closely associated with different forms of aggressive cancer [5,6].

Salivary glands produce saliva, which contains glycoproteins called mucins. There are two classes of mucins: membrane-bound mucins (MUC1 and MUC4) and secreted mucins (MUC2, MUC5AC, MUC5B, MUC6, and MUC7), both synthesized by several types of secretory epithelial cells [4]. Essentially, mucins are high molecular mass glycoproteins that are built up from a central protein core as the backbone (mainly proline, serine/threonine), which is heavily glycosylated (up to 40–80% of the mass consists of O-linked oligosaccharides) with sialic acids on their non-reducing ends (sialylation). These glycoproteins are capable of forming oligomers, while others are monomeric. The most commonly reported genes for salivary mucins are MUC1, MUC5B, and MUC7 [7].

Over the last years, infrared (IR) spectroscopy gained more importance as a non-destructive, label-free, and molecular vibrational spectroscopic technique in the analysis of the biochemical characteristics of tissue samples of various origins, e.g., fingernails, kidney tissue sections, retinal sections, and prostate sections [8,9,10,11]. NIR spectroscopy (spectral range of 780–2500 nm of the electromagnetic spectrum) is a spectroscopic technique with good sample penetration and is based on the absorption of IR radiation of the studied molecular covalent bonds [12]. The (N)IR spectrum arises from the variation of vibrational frequencies of the chemical bonds present in the tissue section. Variation in chemical structure is translated into IR spectral variation. The absorbance bands (overtones) present in the NIR spectra arise from vibrations of molecular bonds within the sample, specifically O–H, C–H, N–H, and S–H bonds [12,13].

The analysis of IR spectra requires more specific statistical techniques based on pattern recognition methods, which are plenty and have proven their worth many times before. The most frequently used methods include principal component analysis (PCA), hierarchical cluster analysis (HCA), and soft independent modeling of class analogy (SIMCA) [14,15,16,17]. Applying these unsupervised (PCA and HCA) and supervised (SIMCA) methods on the NIR spectra of tissue samples render it possible to unravel their fundamental structural biochemistry [8,9,10,11].

In this paper, an attempt was made to explore the potential of NIR spectroscopy to identify a biochemical signature of SGTs on H&E tissue sections and to compare these findings to the current histological classification as the current gold standard.

## 2. Materials and Methods

### 2.1. Study Population 

The present study was approved by the local ethics committee (Ghent University Hospital BC-07309). The authors complied with the World Medical Association Declaration of Helsinki regarding ethical conduct in research involving human subjects.

A total of 238 unique H&E tissue sections of salivary glands (117 males and 121 females) were examined with NIR spectroscopy. Demographic characteristics of the study population can be found in Table 1. From the period of 2015 until 2020, 186 patients (90 males and 96 females; median age: 59 years; IQR: 51–69 years) with SGTs and tissue sections of 52 patients (27 males and 25 females; median age: 56 years; IQR: 48–68 years), who received surgery for non-tumoral disease (control group), were included. 

The study population contained control samples (no tumor) and 11 histological tumor classes: pleomorphic adenoma (myxoid, mixed, and cellular), Whartin tumor, oncocytoma, acinic cell carcinoma, mammary analogue salivary carcinoma, epithelial myoepithelial carcinoma, adenoid cystic carcinoma, mucoepidermoid carcinoma, and salivary duct carcinoma. In total, 220 salivary tissue sections originated from the parotid gland in which 16 were from the submandibular gland, and 2 were from minor salivary glands.

### 2.2. Preparation of Tissue Sections

For the tissue sections, all salivary gland tissues obtained through surgery were fixed with 10% neutral-buffered formalin for 6–48 h. After fixation, salivary gland samples were routinely processed using a Tissue-Tek^®^ VIP^®^ (Sakura, Torrance, CA, USA), after which they were embedded in paraffin. Subsequently, 4 µm tissue sections were cut and stained with H&E. Tissue sections were subjected to standard histological examination. NIR spectroscopic analysis was performed on archived H&E tissue sections. 

### 2.3. Histological Classification

Detailed histopathologic classification of the H&E SGT tissue sections was performed according to the criteria of the current WHO classification. The classification of the cases was confirmed by an experienced head and neck pathologist [1].

The following tumors were classified, according to the WHO classification, as benign tumors: pleomorphic adenoma (myxoid, mixed, and cellular variants), Whartin tumors, and oncocytoma [1]. If the tissue section of the pleomorphic adenoma had a predominant myxoid component, a diagnosis of myxoid type was made; if the cellular component was <50%, the tumor was classified as mixed; and if the cellular component was >50%, a diagnosis of a cellular type was rendered. This classification was performed arbitrarily by two investigators (R.C. and D.C.).

Acinic cell carcinoma, mammary analogue salivary carcinoma, adenoid cystic carcinoma, epithelial-myoepithelial carcinoma, and mucoepidermoid carcinoma were classified as malignant. Squamous cell carcinoma, as a metastasis from scalp skin lesions or ex pleomorphic adenoma, was excluded.

### 2.4. NIR Spectroscopical Analysis

For spectral analysis, a NIR spectrometer, equipped with extended indium gallium arsenide (InGaAs) array technology, was used (AvaSpecNIR256-2.5-HSC, Avantes, Apeldoorn, the Netherlands). The tissue sections were analyzed in ambient temperature and in batches in order to minimize external variabilities, according to the previously described procedure [8]. A white reference tile was used as a base platform and was layered with immersion oil to eliminate loss of resolution due to different refractive surfaces (glass versus air) and immobilized. A 50 mm integrating sphere (AvaSphere-50-LS-HAL-6-S1, Avantes) with a 6 mm sample port diameter was placed stably and directly onto the tissue sections for analysis. Analysis was performed in the salivary gland zone, indicated by a pathologist after microscopic analysis as histologically distinctive for the SGT. This zone was indicated on the tissue section with a marker. Spectra of the reflected light were recorded across the range of 1000–2400 nm at a resolution of 13 nm. A total of 128 spectra were made and averaged. The procedure was repeated one time for each SGT tissue section.

### 2.5. Neuraminidase Treatment of Tissue Sections

Representative paraffin tumor tissue blocks of 45 randomly selected SGTs (9 controls, 5 pleomorphic adenomas, 5 Whartin, 5 adenoid cystic carcinoma, 5 acinic cell carcinoma, 5 epithelial myoepithelial carcinoma, 6 mucoepidermoid carcinoma, and 5 salivary duct carcinoma) were collected from the authors’ institutional archives. For each case, 8 µm thick sections were cut and deparaffinized.

α-2-3,6,8 Neuraminidase (Acetyl-neuraminyl hydrolase and sialidase) catalyzes the hydrolysis of α2-3, α2-6, and α2-8 linked N-acetyl-neuraminic acid residues from glycoproteins and oligosaccharides. Neuraminidase (2000 units, ref P0720S, New England Biolabs, Ipswich, MA, USA) and the corresponding solvent solution were mixed and diluted with saline 1/10, according to company guidelines. A droplet of 250 µL (200 u/mL) was applied onto one series of the deparaffinized tissue sections (post-neuraminidase group). On another series of tissue sections, only phosphate-buffered saline (PBS) (0.1 mol/L, containing 137 mmol/L NaCl, 2.7 mmol/L KCl, 8.1 mmol/L Na_2_HPO_4_, and 1.47 mmol/L KH_2_PO_4_, pH 7.3) (250 µL) was placed and acted as a control group (pre-neuraminidase group). Subsequently, all tissue sections were incubated (24 h, 37 °C). Finally, the tissue section was washed using saline water and left to air dry for 6 h (ambient temperature).

### 2.6. Data Analysis

#### 2.6.1. Data Preprocessing

The spectral data were exported from the NIR spectrometer software (Aventes) (*n* = 238). Preprocessing, PCA, and SIMCA analyses were performed using SIMCA software version 16.0.2.10561 (Sartorius Stedim Data Analytics(R), Goettingen, Germany). All individual spectral data (an average of 128 scans) were loaded and analyzed after preprocessing according to the principles of PCA and SIMCA as described below.

All samples were scanned in the NIR region from 1000 to 2400 nm. Spectral data from the H&E tissue section-based SGTs were mean-centered, normalized using the standard normal variate method (SNV), and converted to their first and second derivatives with a Savitzky–Golay (S-G) algorithm (9 smoothing points).

#### 2.6.2. Principal Component Analysis (PCA)

PCA is a projection method that allows the projection of high-dimensional data into a low-dimensional space. This low-dimensional space is defined by new orthogonal latent variables, commonly referred to as principal components (PCs). The result of such an analysis is a reduction in the number of variables by calculating linear combinations (=PCs) of these original variables. The first constructed PC represents the highest variance in the data; the second PC explains the highest residual variance, around the first PC. Hence, this second PC is by definition orthogonal to the first. PCA projects each object (=sample) on the created PCs. These projections are referred to as scores and provide information about the (dis)similarities among the objects. PCA was used to illustrate differences between all of the studied material and to explore whether this technique can result in a clustering of samples, which is useful for the creation of classification models [18,19].

#### 2.6.3. Soft Independent Modelling of Class Analogy (SIMCA)

By using supervised (modeling) techniques (SIMCA) and the significant wavelength numbers of the neuraminidase treatment of the tissue sections, classification models could be built. First, the dataset of the population, containing the control group and malignant SGTs, was randomly divided into training (65% of the samples) and test sets (35% of the samples). The same procedure was repeated three times with different compositions of both sets in order to ensure the inclusion of all samples in the test set at least once [18,19].

### 2.7. Statistical Analysis

Statistical analysis was carried out using MedCalc Version 18.11 (MedCalc Software, Mariakerke, Belgium). Wilcoxon signed ranked tests were used to find statistically significant differences in NIR wavelengths between pre-treatment and post-treatment with neuraminidase (paired variables). The differences between the intensity of the first derivative in the NIR spectra showed the variation in spectral changes due to the reaction with neuraminidase, graphically depicted in box-and-whiskers plots. A *p*-value < 0.05 was considered a priori to be statistically significant.

## 3. Results

### 3.1. Descriptive Spectral Analysis of SGTs

**Figure 1:**
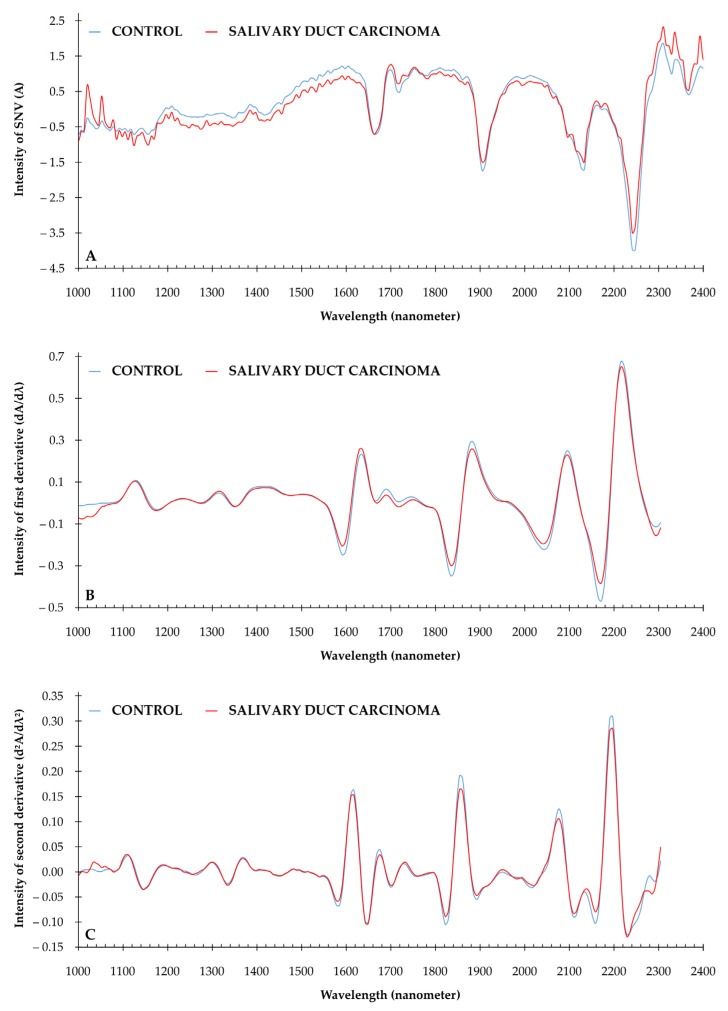
Median NIR spectra for control group and salivary duct carcinoma. Spectral range from 1000 to 2400 nanometer. (**A**) NIR spectra after preprocessing SNV; (**B**) NIR spectra after SNV and S-G smoothing (9 smoothing points) and 1st derivative; (**C**) NIR spectra after SNV, S-G and 2nd derivative.

Figure 1 illustrates the NIR spectral data of a control sample and a sample of salivary duct carcinoma. The preprocessed NIR spectra are presented for the spectra according to standardized normalized variates (SNV; Figure 1A); according to SNV, S-G algorithm (9 smoothing points), and first derivative (Figure 1B); and according to SNV, S-G algorithm (9 smoothing points), and second derivative (Figure 1C).

**Figure 2:**
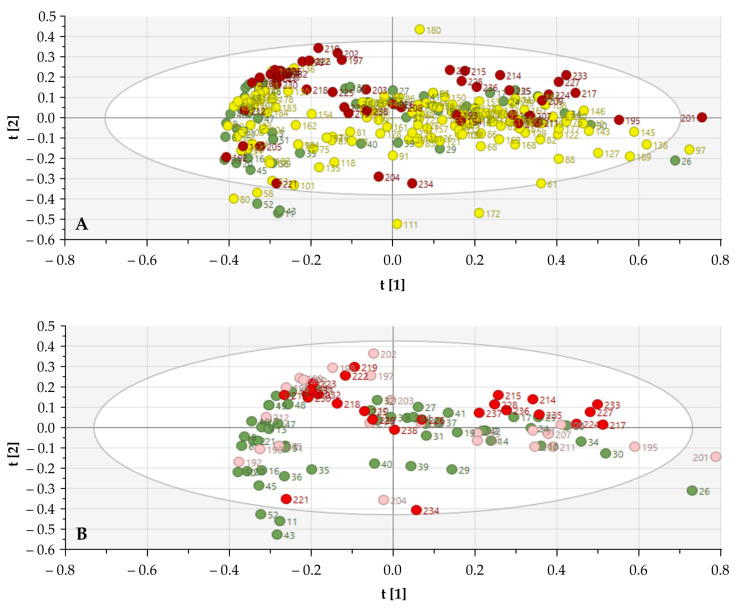
Score plots of all NIR spectra for differentiation of salivary gland specimens. (**A**) Score plot of all tissue samples according to the oncological classification (control (green), benign (yellow), and malignant (red)). (**B**) Score plots according to malignant histological classification (control (green); acinic cell carcinoma, mammary analogue salivary carcinoma, and adenoid cystic carcinoma (light red); and epithelial myoepithelial carcinoma, mucoepidermoid carcinoma, and salivary duct carcinoma (dark red)).

The NIR spectra of 238 SGTs were analyzed using PCA. Initially, potential differences were identified by comparison of the SNV normalized, first derivative, and SG smoothed (9-11-15 points) spectra between the different histological classifications of SGTs. Due to the large amount of data and classes, no clear differences could be observed between the NIR spectra of the SGTs, indicating the need for a reduction in the number of histological tumor classes. The SGTs were consequently categorized according to their oncological severity. Large groups were made: control group (*n* = 52), benign SGTs (pleomorphic adenoma, Whartin, and oncocytoma), and malignant SGTs (acinic cell carcinoma, mammary analogue salivary carcinoma, adenoid cystic carcinoma, epithelial myoepithelial carcinoma, mucoepidermoid carcinoma, and salivary duct carcinoma). Using PCA, the clustering of malignant SGTs became clearer. A total of 0.74 of the total variation could be explained by using the first two PCs. SIMCA analysis of the entire NIR spectra (all wavelengths) generated a correct classification ratio of 73.1% (control 78.4%, benign SGTs 64.3%, and malignant SGTs 92.17% correct) (Figure 2).

### 3.2. Neuraminidase Treatment of Tissue Sections—Spectral Analysis

**Figure 3:**
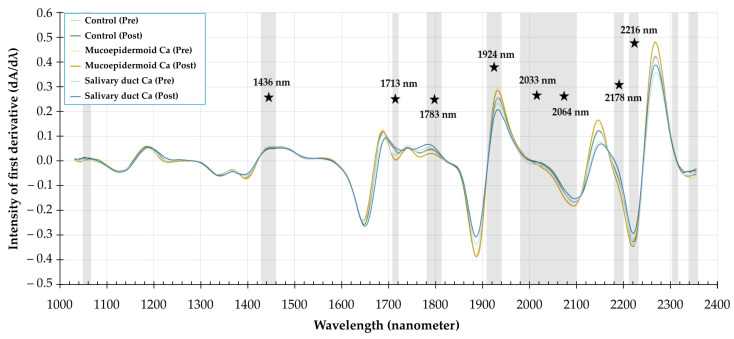
Discriminative spectral features in SGTs after neuraminidase treatment. The first derivatives of the median NIR spectra of SGT tissue sections pre-treatment (pre) and post-treatment (post) with α-2-3,6,8 neuraminidase are shown. Grey boxes on the spectra indicate the statistically significant wavelengths between the pre-and post-treatment group (Wilcoxon signed ranked test). The star symbols mark the position of most statistically significant wavelengths in the NIR spectrum. Ca, carcinoma.

A series of deparaffinized tissue sections of SGTs (*n* = 45) were treated, respectively, with PBS (pre-treatment) as a control group and neuraminidase (post-treatment) as an experimental group. Figure 3 illustrates the first derivative of the median pre-treatment and post-treatment NIR spectra of the control group (*n* = 9), mucoepidermoid carcinoma (*n* = 6), and salivary duct carcinoma (*n* = 5). Statistically significant differences were found in several spectral regions: 1052–1065 nm; 1430–1462 nm; 1713–1719 nm; 1777–1809 nm; 1911–1943 nm; 1988–2108 nm; 2178–2197 nm; 2209–2216 nm; 2304–2311 nm; and 2342–2349 nm. These regions are marked with a grey box. The most significant wavelengths, marked with a star symbol, were 1436 nm (*p* = 0.0006), 1713 nm (*p* = 0.035), and 1783 nm (*p* = 0.018); 1924 nm (*p* = 0.0021) and 2032 nm (*p* = 0.0027); 2064 nm (*p* = 0.0042), 2178 nm (*p* = 0.015), and 2216 nm (*p* = 0.039). With relevant wavelengths identified, we proceeded to evaluate the potential of NIR spectroscopy for detecting SGTs by applying SIMCA.

**Figure 4:**
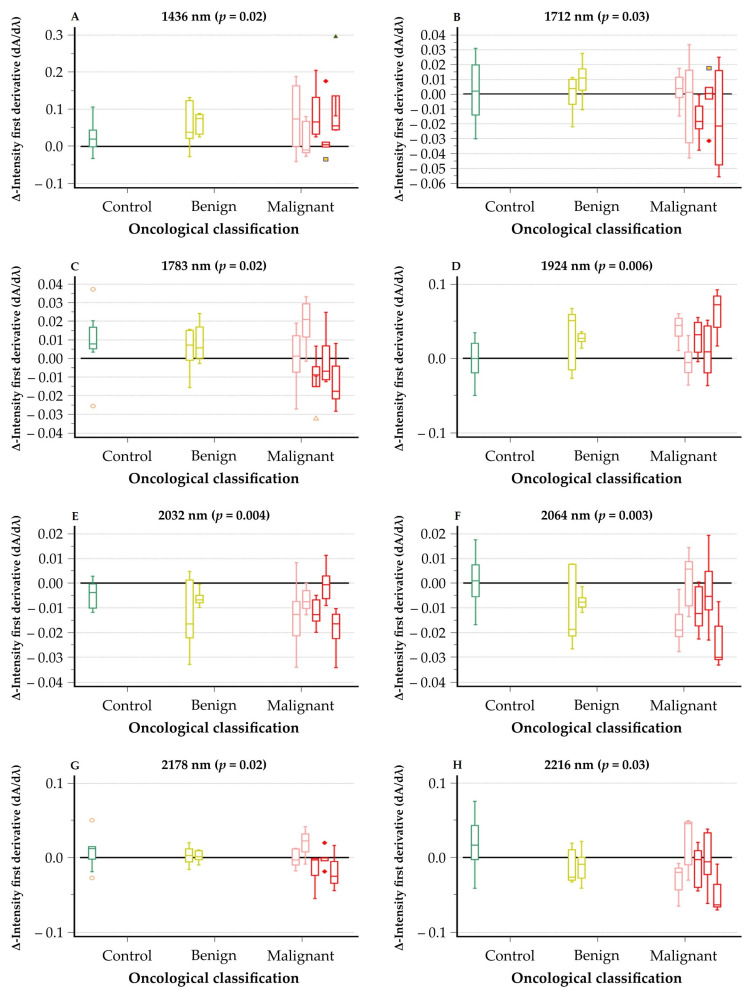
Box-and-Whiskersplot of Δ-intensity of SGT for most significant NIR wavelengths with corresponding p-value: (**A**) wavelength 1436 nm; (**B**) wavelength 1712 nm; (**C**) wavelength 1783 nm; (**D**) wavelength 1924 nm; (**E**) wavelength 2032 nm; (**F**) wavelength 2064 nm; (**G**) wavelength 2178 nm; (**H)** wavelength 2216 nm. All SGTs listed from left to right are as follows: control group (green); pleomorphic adenoma (myxoid, mixed, and cellular variant; yellow) and Whartin tumor (yellow); acinic cell carcinoma (light red) and adenoid cystic carcinoma (light red); epithelial myoepithelial carcinoma (dark red), mucoepidermoid carcinoma (dark red), and salivary duct carcinoma (dark red). Significance has been indicated in the figure.

The intensity differences in the first derivative of the spectral signal according to aforementioned wavelengths (Figure 4) demonstrated a more significant change in spectral signal of the epithelial myoepithelial carcinoma, mucoepidermoid carcinoma, and salivary duct carcinoma due to the treatment with neuraminidase. This phenomenon is not observed with acinic cell carcinoma, adenoid cystic carcinoma, and benign tumors. Although this trend is not always the case for every wavelength, it strongly suggests the effect of neuraminidase on the mucins present in the SGTs.

### 3.3. Discriminative Power of the Biochemical Signature

**Figure 5:**
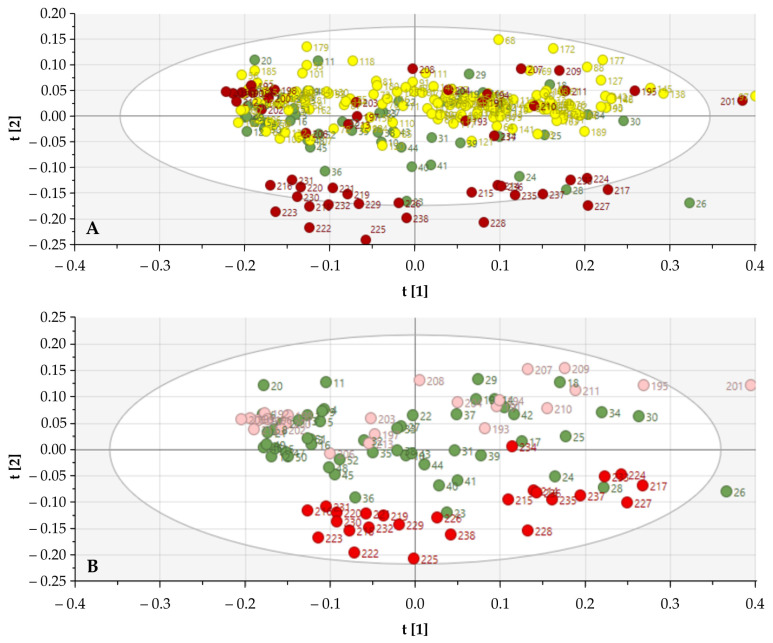
Score plots of NIR spectra following neuraminidase treatment for differentiation of salivary gland specimens. The effect of narrowing to the region of interest in the NIR spectra (according to the wavelengths mentioned in Figure 3) is illustrated. A clear difference can be noted in comparison to the previous score plot (Figure 2). (**A**) Score plot of all tissue samples according to the oncological classification (control (green), benign (yellow), and malignant (red)). (**B**) Score plots according to malignant histological classification (control (green); acinic cell carcinoma, mammary analogue salivary carcinoma, and adenoid cystic carcinoma (light red); and epithelial myoepithelial carcinoma, mucoepidermoid carcinoma, and salivary duct carcinoma (dark red)).

Guided by the statistically significant regions after neuraminidase treatment, the PCA model showed enhanced clustering of the malignant SGTs tumor, with the exception of adenoid cystic carcinoma and acinic cell carcinoma. The first two PCs explained 0.88 of the total variation. One malignant sample (mucoepidermoid carcinoma, subject 234) and three control samples (subject 23, 24, and 28) were misclassified (Figure 5). SIMCA analysis based on only the significant NIR regions generated a correct classification ratio of 91.9%. Clear clustering was found between the control group and mucoepidermoid carcinoma, epithelial myoepithelial carcinoma, and salivary duct carcinoma. No differences were observed between benign tumors or acinic cell carcinoma and adenoid cystic carcinoma.

When constructing a new model between the control group and epithelial myoepithelial carcinoma, mucoepidermoid carcinoma, and salivary duct carcinoma, an almost perfect separation between the control and tumor group could be found. SIMCA analysis found a correct classification ratio of 97.1% between the control group and the malignant group (epithelial myoepithelial carcinoma, mucoepidermoid carcinoma, and salivary duct carcinoma).

## 4. Discussion

Histological microscopic analysis and immunostaining of SGTs, still seen as the standard procedure for diagnosing SGTs, are difficult due to its complexity and rarity, often resulting in additional imaging (ultrasound, FNAC, CT-scans, or MRI-scans) [2,3,20]. According to the present study’s results, NIR spectroscopy, clarifying also the biochemical constitution of the tumor, could be beneficial for identifying malignancy in SGT’s.

Some authors investigated saliva and its relation to oral pathology with IR spectroscopy. One group described the role of saliva in the early diagnosis of SGTs using Attenuated total reflection Fourier-transform infrared spectroscopy (ATR-FTIR) [21]. Raman spectroscopy and imaging seem to be suitable for characterizing noncancerous and cancerous tissues in saliva [22]. FTIR microspectroscopy on the mouth, salivary glands, and oral cystic lesions objectively discriminates normal from dysplastic and cancer states characterizing also the grading [23]. The first analysis of glycoproteins, such as mucins, was already performed by using FTIR. It was shown that sugars are found in saliva in a form bound with proteins, probably referring to glycoproteins such as mucins [24,25]. A disadvantage of FTIR is the time needed for sample preparation. Raman spectroscopy, unlike FTIR, is faster in preparing samples [21,26,27,28].

In the present study, the H&E tissue sections were processed following a fixed protocol and were equal in thickness, resulting in reproducible NIR spectra. The total time cost to analyze one tissue section with NIR was less than one minute.

This method is not restricted to very specialized laboratories and can be performed on routinely stained tissue sections without the need for additional sample preparation. This technique has future perspectives for rapid, simple, and automated diagnosis or screening that is nearly independent of the operator.

Nevertheless, NIR as a novel technique in SGT diagnosis also has some limitations. Firstly, SGTs are very heterogeneous tumors. Thus, analysis of only a small fraction of the tissue section can result in changes in the NIR spectra with misclassification as a consequence. This can be countered by analyzing multiple fragments of the same SGT. Secondly, NIR spectroscopy on SGTs only provides a rather general insight into tissue biochemistry, in contrast to the histological microscopical investigation. Thirdly, NIR spectroscopy is based on the absorbance at specific wavelength(s) and can be affected by the thickness and particle size of the sample, by variations of the optical path length, and by crystalline forms, requiring well-defined sample analysis preparation protocol [12,14]. Fourthly, the deparaffinization of the tissue sections (neuraminidase experiment) could have structurally changed the tissue sections, which result in alterations in the NIR spectra and could differ from the analysis obtained with the H&E tissue sections. Finally, formalin fixation can have an influence on NIR spectral measurements. Therefore, no fresh tissue sections were subjected to NIR spectroscopy in order to reduce possible confounding factors. All of these disadvantages were countered as much as possible by using a fixed analysis protocol as described above.

Some authors investigated the enzymatic effect on the structure of MUC5B. The predominant enzymatic activities were shown to be glucosidases and β-N-acetyl-glucosaminidase with additional strong reactivity of α-l-fucosidase, β-galactosidase, β-N-acetylgalactosaminidase, neuraminidase, and α-l-fucosidase. The latter two are of special interest because terminal fucosylated and sialylated structures are commonly found on MUC5B oligosaccharides. No activity was found for α-arabinosidase, β-fucosidase, and α-galactosidase [29,30,31,32].

The present study suggests that mucins could be substrates for the applicated neuraminidase. It is difficult to understand where exactly the enzymatic reactions with neuraminidase happened in the tissue sections (intra-or extracellular) or what the clinical relevancies of these desialysations are.

Furthermore, this study demonstrates promising prospects for thorough enzymatic dissection of SGT mucins with other types of enzymes, resulting in further insights of the biochemical composition of the SGTs.

## 5. Conclusions

The current study aimed to explore the potential of NIR spectroscopy for identifying a biochemical signature of SGTs on H&E stained tissue sections and to compare these findings to the current histological classification. Using a neuraminidase experimental approach, it was possible to distinguish malignant SGTs from benign tumors based on spectral peak differences. Although the technical implementation still has room for improvement and other enzymatic treatments are possible, this study is one of the first to investigate SGTs with NIR spectroscopy in this manner. This study presented some remarkable ideas that can improve the classification of rare and histologically difficult tumors in general. We can conclude that H&E tissue section-based NIR spectroscopy can identify malignant SGTs from a healthy control group, resulting in a promising alternative method in the diagnosis of SGTs.

## Figures and Tables

**Table 1:** Demographics characteristics of the study population.

Study Population	All		Male		Female	
	*n*	Age (yrs)	*n*	Age (yrs)	*n*	Age (yrs)
Total	238	59 (50–69)	117	58 (51–69)	121	59 (48–68)
Control group	52	56 (48–68)	27	61 (49–70)	25	55 (48–64)
Salivary Gland Tumours	186	59 (51–69)	90	58 (51–69)	96	60 (51–69)
Benign						
Pleomorphic adenoma	79	55 (47–63)	37	52 (44–67)	42	56 (48–62)
✓ Myxoid	40	55 (46–62)	17	54 (44–62)	23	55 (47–62)
✓ Mixed	7	59 (48–62)	4	54 (49–65)	3	60 (48–62)
✓ Cellular	32	55 (47–67)	16	52 (44–71)	16	56 (48–62)
Whartin tumour	53	63 (57–71)	31	63 (56–71)	22	63 (59–72)
Oncocytoma	5	64 (57–81)	2	70 (61–79)	3	64 (51–81)
Malignant						
Acinic cell carcinoma	11	55 (53–61)	3	53 (50–57)	8	56 (54–65)
Mammary Analogue Salivary Carcinoma	4	66 (62–75)	2	66 (62–69)	2	71 (62–80)
Epithelial Myoepithelial Carcinoma	10	57 (51–72)	4	54 (38–69)	6	63 (52–72)
Adenoid Cystic Carcinoma	9	61 (57–67)	6	60 (53–66)	3	69 (62–71)
Mucoepidermoid carcinoma	9	57 (54–67)	2	53 (52–54)	7	57 (55–76)
Salivary Duct Carcinoma	6	59 (56–62)	3	58 (57–60)	3	62 (56–64)

Data for age are given as median (interquartile range); yrs, years.

## Data Availability

The data presented in this study are available upon request from the corresponding author. The data are not publicly available due to European General Data Protection Regulation.
